# Recent Advances in Conjugated Graft Copolymers: Approaches and Applications

**DOI:** 10.3390/molecules24163019

**Published:** 2019-08-20

**Authors:** Tomasz Jarosz, Karolina Gebka, Agnieszka Stolarczyk

**Affiliations:** 1 Department of Physical Chemistry and Technology of Polymers, Silesian University of Technology, 9 Strzody Street, 44-100 Gliwice, Poland; 2 Department of Inorganic Chemistry, Analytical Chemistry and Electrochemistry, Silesian University of Technology, 6 Krzywoustego Street, 44-100 Gliwice, Poland

**Keywords:** graft copolymer, conjugated polymer, polyaniline, polypyrrole, polythiophene, grafting

## Abstract

The main goal of this mini review is to summarise the most recent progress in the field of conjugated graft copolymers featuring conjugation across the main chain, across side chains or across both. The main approaches to the synthesis of conjugated graft copolymers are highlighted, and the various trends in the development of new copolymer materials and the intended directions of their applications are explored.

## 1. Introduction

Graft conjugated macromolecules consist of numerous arms attached to an oligomeric or polymeric core, with either the arms, the core or both being conjugated. Depending on whether the core is conjugated or not, the arms can interact electronically with each other or are isolated from each other. Although no direct conjugation route may exist between the neighbouring arms, they can interact with each other through the formation of π-stacks or ordered assemblies, similar to what is observed for bulk linear polymers [1]. The key parameters of such macromolecules are the chemical structure and length (usually expressed as the degree of polymerisation) of the core and arms, as well as the grafting density and distribution of arms along the core.

The synthesis of graft conjugated systems is possibly the most varied of the three mentioned types of 3D-structured conjugated polymers, as it encompasses the methods used for the preparation of star-shaped (mainly focusing on linking arms and cores—grafting “onto”) and dendrimeric (mainly functionalization and branching at active sites—grafting “from”) systems, as well as a variety of other methods, including reactive assembly of the core through the polymerisation of macromolecular monomers (grafting “through”):

- Grafting “through”—typically includes anionic, free radical polymerisation and ring-opening metathesis, and it is polymerisation from macromonomers. If graft copolymers are produced via this approach, each repeat unit of the copolymer main chain has polymeric side chains, but the distance between them may differ depending on the methods that were used. As an example, in ring-opening metathesis where polynorbornene is used as a backbone, there are four carbon atoms between side chains, and with the use of polymethacrylate there are only two carbon atoms.
- Grafting “onto”—in this method backbone and side chains are prepared separately. This approach can be associated with two potential issues—limited grafting density and how to remove unreacted side chains.
- Grafting “from” allows high grafting density and long backbones to be obtained; in this category, reversible deactivation radical polymerisation, atom transfer radical and reversible nitroxide-mediated polymerisation can be used. These structures could potentially be used in drug delivery systems, as super-soft elastomers, surfactants, lubricants, stimuli-responsive materials, etc. [2].

**Scheme 1:**
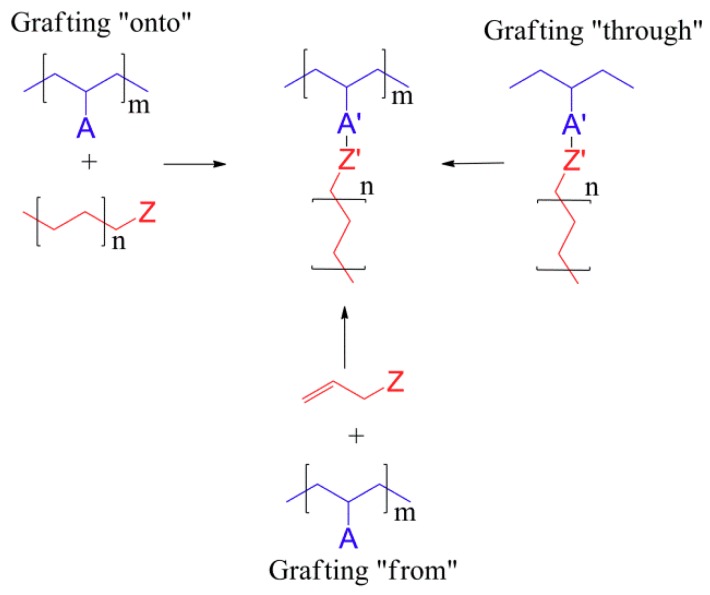
Outline of the three main approaches to the synthesis of graft polymers. Based on [4]. The A and Z groups are used to indicate the reactive groups in each type of reaction. The example of polyethylene grafting was selected rather than the species reported in this work in order to maintain clarity and focus on the reactions that may be used for obtaining these macromolecules.

The general schemes for the three main classes of synthetic pathways to graft conjugated polymers are shown in Scheme 1. In terms of particular synthetic procedures, Bousquet et al. [3] reviewed a wide array of synthetic methods. Even though the authors focused on the synthesis of polymer brushes on substrate surfaces rather than on the development of graft macromolecules, the procedures for producing polymer brushes on organic substrates are of interest, with many having been adapted or used directly to produce graft polymers.

The progress in the field of designing and synthesising graft conjugated polymers was recently summarised by Strover et al. One highlight of this work is the conclusion that the utility of graft copolymers with conjugated backbones could be improved by modifying their electroactivity or solubility with side chains. The chemical methods for synthesising copolymers are better for preparing large amounts of compounds, and are preferable for optoelectronic devices. The electrochemical method is instead useful for modifying surfaces. The choice of the copolymerisation approach is dependent on what applications and structure are desired. Grafting “from” is generally used for preparing densely grafted copolymers. With grafting “through”, well-defined and highly sophisticated structures can be produced. Lastly, grafting “onto” is possibly the most straightforward approach, because of the highly versatile click reactions employed [5].

Yassar et al. [6] have also included a number of graft polymers in their review of block copolymers used for photovoltaic applications. The issues related to the control of the morphology of any produced polymeric layers are highlighted, and the need for better understanding of the various process parameters is emphasised. Consequently, only the most recent advances in the field are summarised below, with a focus on potential optoelectronic applications of the reported macromolecular systems.

## 2. Recent Developments and Applications of Graft Copolymers

### 2.1. Basic Research on Graft Copolymers

**Scheme 2:**
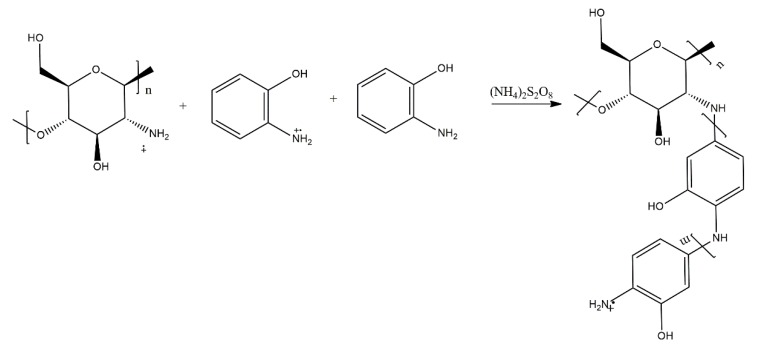
Grafting of poly(2-hydroxyaniline) on chitosan by El-Salam et al. in [7].

Abd El-Salam et al. [7] report the grafting of poly(2-hydroxyaniline) on chitosan (Scheme 2). The authors investigated the effect of different copolymerisation conditions on the properties of their copolymers, confirming their structure via infrared (IR) spectroscopy. The mechanism and kinetics of the grafting copolymerisation reaction are discussed in great detail. The samples of the copolymers were investigated in terms of their adsorption parameters. These materials show favourable adsorptive properties towards removing transition metals from aqueous media. It however remains unclear whether the materials are true copolymers or a blend of homopolymers produced via polymerisation of the monomer adsorbed on chitosan particles.

**Scheme 3:**
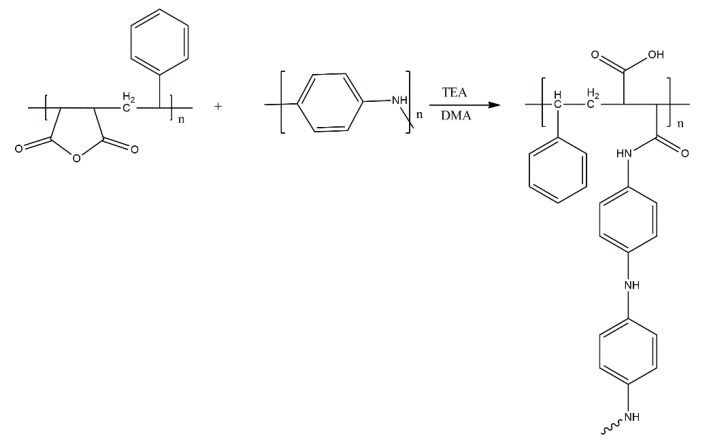
Grafting of polyaniline (PANI) on poly(styrene-alt-maleic anhydride) (PSMA) by Heydari et al. [8].

Heydari et al. [8] (Scheme 3) proposed a method to obtain water-soluble polyaniline (PANI) copolymers by grafting poly(styrene-alt-maleic anhydride) with PANI, yielding conductive comb copolymers (PANI-g-PSMA) with highly improved processability. First, PANI nanoparticles were prepared by chemical synthesis under ultrasonic irradiation. Then the poly(styrene-alt-maleic anhydride) (PSMA) was synthesised by free-radical polymerisation. Concurrently, PANI was grafted on the PSMA backbone to prepare a comb-like conductive copolymer for improving its processability, as a new method.

**Scheme 4:**
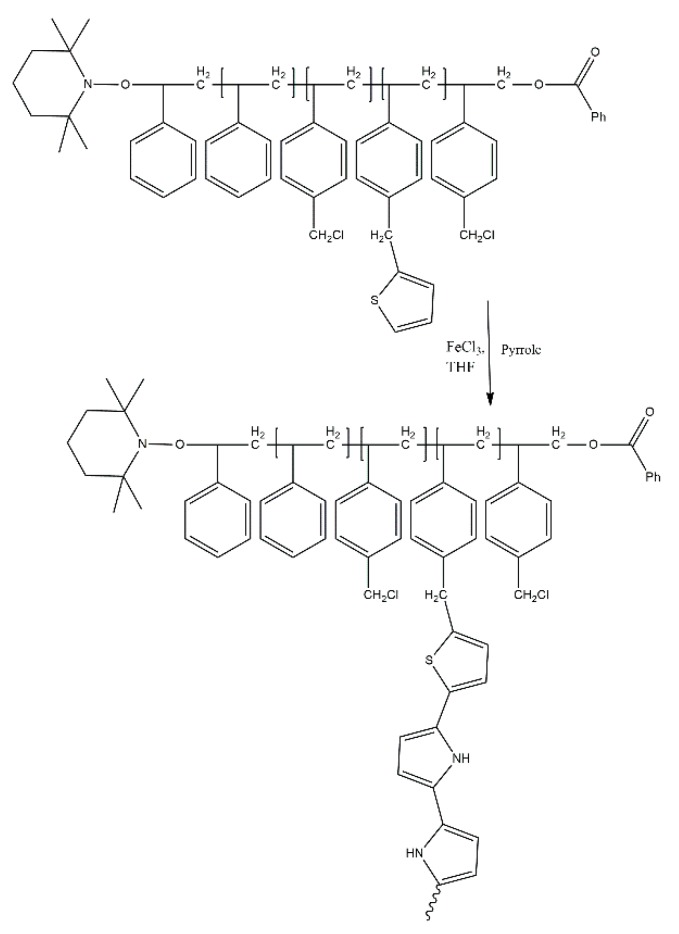
Grafting PPy onto Th-functionalised polystyrene (PS) by [9].

Another interesting report [9] deals with the synthesis of polypyrrole (PPy) grafted onto a thiophene-functionalised polystyrene (PS) macromonomer via the chemical oxidative polymerisation of pyrrole in the presence of the macromonomer (Scheme 4). Hatamzadeh et al. employed a very thorough product purification methodology, involving the sequential dissolution of non-grafted polymer chains. First, non-grafted polystyrene was dissolved in cyclohexane and then PPy was dissolved in tetrahydrofuran (THF), leaving only the graft copolymer, which was insoluble in both these solvents; this result can also be considered as evidence for the occurrence of copolymerisation. The growth of PPy on the functionalised PS enhanced the solubility and processability of the copolymer, when compared with PPy. Despite its linkage to a non-conjugated system, the PPy segments maintained good redox activity and overall, the synthesised graft copolymer was found to show acceptable stability of its redox properties.

**Scheme 5:**
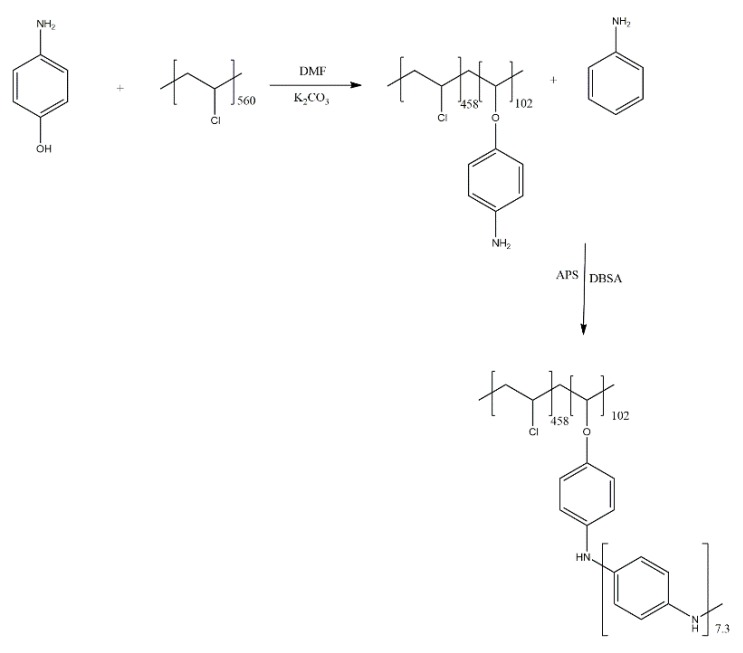
Grafting of PANI on poly(vinyl chloride) (PVC) [10].

In another variation of their approach, Massoumi et al. investigated the grafting of PANI on poly(vinyl chloride) (PVC) [10]. The chemically obtained aniline-functionalised PVC macromonomer was employed in a mixture with aniline and polymerised both chemically and electrochemically (Scheme 5). The chemical structure of both types of graft copolymers were confirmed by IR and nuclear magnetic resonance (NMR) spectroscopy, with the copolymers being investigated electrochemically and via conductometry. Expectedly, the graft copolymers were found to exhibit lower electrical conductivity as lesser electroactivity than PANI.

### 2.2. Graft Conducting Polymer Hydrogels

Conducting hydrogels are gels that, upon contact with water, absorb it and undergo swelling, while maintaining their conductive properties. Most of this type of hydrogel contain polyaniline, polypyrrole, or poly(3,4-ethylenedioxythiophene) (PEDOT), fulfilling the role of conductive segments. There are also more complex structures, such as gelatine-grafted polyaniline, carboxymethylchitosan, oxidised dextran or polyaniline hydrogels with poly(acrylic acid), grafted with gelatine, and alginate. Even though these compounds exhibit interesting properties and have many prospective applications, they are difficult to investigate in terms of their structure and performance [11].

**Scheme 6:**
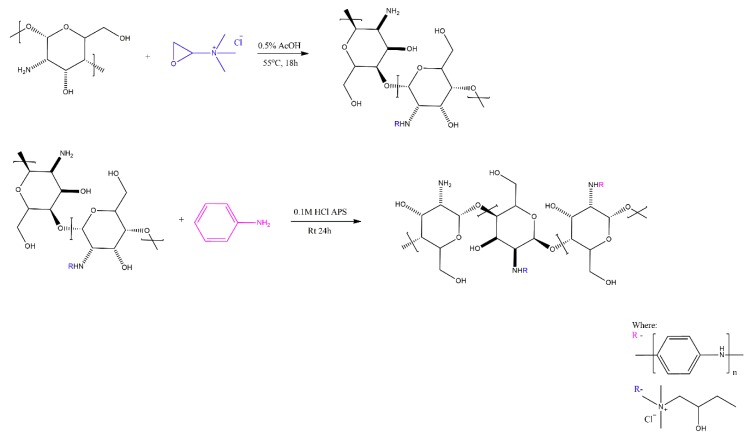
Grafting of glycidyltrimethylammonium and poly(aniline) on chitosan [12].

Guo et al. [12] obtained quaternised (chitosan with grafted glycidyltrimethylammonium chloride) chitosan-grafted-PANI (QCS-g-PANI) by chemical synthesis in aqueous HCl (Scheme 6), in the presence of ammonium peroxydisulphate at room temperature. The degree of grafting was found via gravimetry, as a ratio of the mass of PANI, dissolved away in NMP, to the mass of the introduced monomer. This method may have some issues, as it does not take into account the molecular weight of the attached PANI grafts. These two compounds were used to reduce the toxicity of quaternised chitosan (QCS), while improving its electroactivity by using PANI, and the Authors also hypothesised that the synergetic effect of QCS and PANI would increase antibacterial activity of the obtained hydrogels. The obtained hydrogels showed good antibacterial activity for Gram-positive and Gram-negative bacteria (5% surviving cells for *Escherichia coli* and 10% surviving cells for *Staphylococcus aureus* in vitro, at a bacterial concentration of 10^6^ CFU/mL), for QCS40P3-ODex (6 mg of aniline and 194 mg of QCS40).

**Scheme 7:**
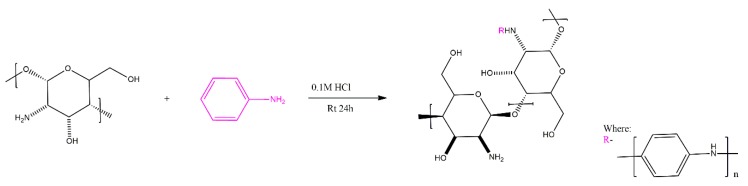
Grafting of PANI on chitosan [12].

Guo et al. have further employed their methodology in order to produce chitosan-g-PANI copolymers with dextran polymer as a cross-linker for use as drug carriers (Scheme 7). The release of the drug amoxicillin was controlled via voltage application, inducing doping/dedoping of the PANI segments interacting with the drug, similar to the approach reported for PEDOT-based drug delivery systems [13,14].

**Scheme 8:**
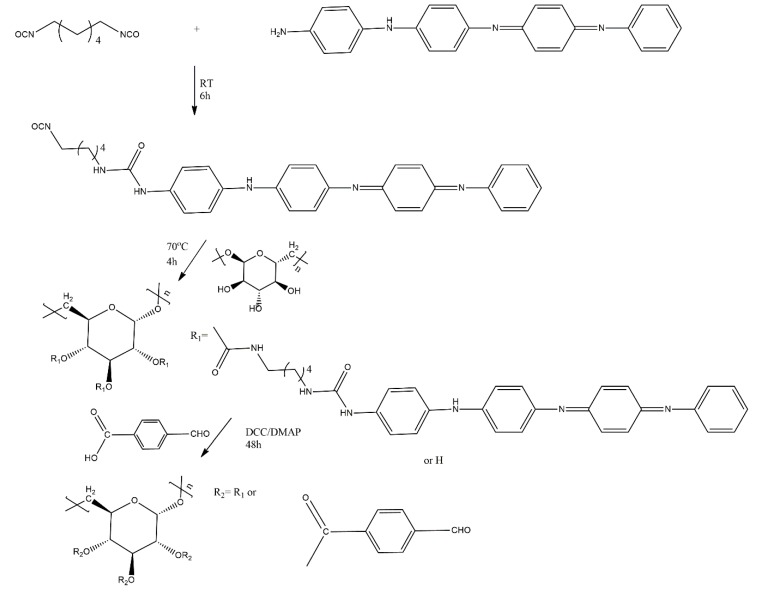
Syntheses of dextran-graft-(aniline tetramer)-graft-(4-formylbenzoic acid) [15].

Guo et al. have also synthesised hydrogels that showed rapid self-healing. These hydrogels consisted of a solution of *N*-carboxyethyl chitosan and synthesised copolymer dextran-graft-(aniline tetramer)-graft-(4-formylbenzoic acid). To synthesise this copolymer, hexamethylene diisocyanate-graft-aniline tetramer was first obtained and subsequently substituted with dextran. Afterwards, 4-formylbenzoic acid was grafted (Scheme 8). The hydrogels showed in vivo (in rats) injectability and in vitro biodegradability, and they indicated self-healing ability because of Schiff base bonds [15].

**Scheme 9:**
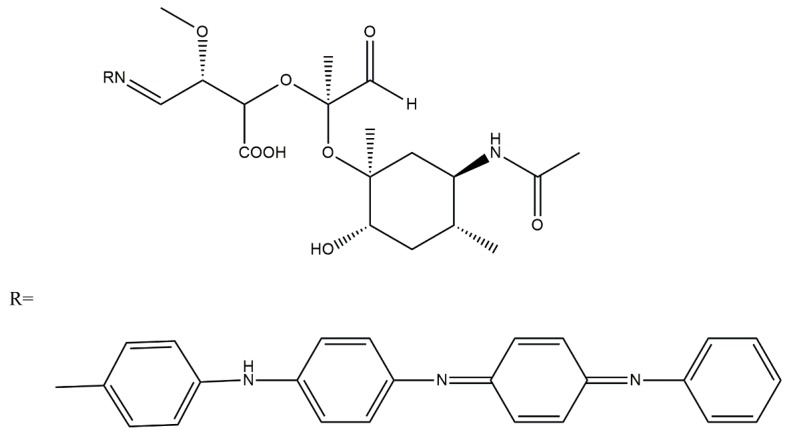
Structure of (oxidised hyaluronic acid)-graft-(PANI tetramer) [16].

In a different attempt, the obtained hydrogels were based on *N*-carboxyethyl chitosan and (oxidised hyaluronic acid)-graft-(PANI tetramer) (Scheme 9) and were designed as injectable drug carriers for delivering amoxicillin (antibiotic). They used IR spectroscopy to confirm the structure of the obtained copolymer and hydrogel. The hydrogels showed good antibacterial properties, helping prevent wound infection. With the addition of aniline tetramer (AT), the healing process of wounds became faster than for hydrogels with copolymers with less grafted aniline tetramer, with higher granulation tissue thickness [16].

Guo and Ma also reviewed the subject of graft copolymers, focusing on systems used in tissue engineering. The biggest disadvantages of conducting polymer are that they are rarely biodegradable. Due to this fact, aniline/pyrrole-based copolymers with hydrolysable groups are becoming increasingly popular. Guo and Ma claimed that they obtained an elastomer based on polylactide, poly(ethylene glycol) (PEG) and aniline trimer which showed a strain at break higher than 1600%. This could be used in skeletal muscle tissue engineering. For example, poly(l-lactide-co-ε-caprolactone) fibres were positive for sarcomeric myosin. Additionally, copolymer can be used for nerve or for skin tissue engineering; PEDOT/chitosan/gelatine had good biocompatibility and improved neuron-like rat cell adhesion and proliferation [17].

**Scheme 10:**
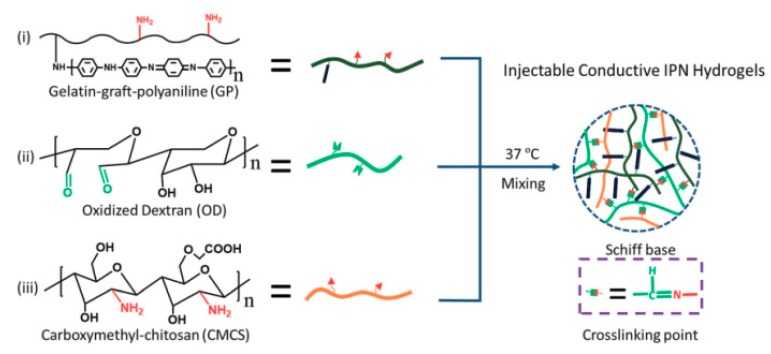
Conductive interpenetrating polymer network (IPN) hydrogel based on gelatine-graft-PANI, oxidized dextran and carboxymethyl chitosan. Reprinted with permission from [18], Copyright 2015, Royal Society of Chemistry.

Li et al. [18] present a simple approach to preparing injectable conductive interpenetrating polymer network (IPN) hydrogels with enhanced mechanical properties. The in-situ IPN-forming conductive hydrogels were based on gelatine-graft-PANI and carboxymethyl chitosan, which were cross-linked with oxidised dextran (Scheme 10). The conductivity, swelling ratio and pore size of the hydrogels were controlled by the PANI content, which was estimated based on gravimetric investigations. The injectable conductive hydrogels showed good cytocompatibility with adipose-derived mesenchymal stem cells, and greatly enhanced the cell proliferation of C2C12 myoblasts. The in vivo (rat) biocompatibility of the hydrogels was also confirmed by subcutaneous implantation, although a mild foreign-body-type immune response was observed upon implantation.

### 2.3. Graft Copolymers for Sensing Applications

**Scheme 11:**
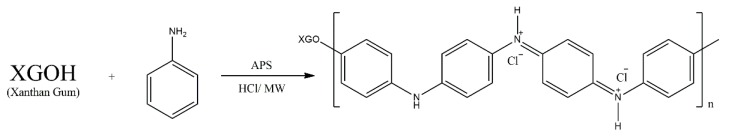
Grafting PANI on xanthan gum.

Pandey and Ramontja [19] report an interesting approach to the modification of natural polymers, with their report on the grafting of aniline units onto xanthan gum (XG) in order to develop a new sensing material (Scheme 11). Grafting was realised via a procedure involving microwave irradiation, with the influence of the irradiation parameters also being studied. They also suggested a mechanism for this grafting, postulating that the first radical is produced through the decomposition of ammonium peroxydisulphate (APS). Simultaneously APS acts as the oxidising agent in the oxidative polymerisation reaction of polyaniline. The last postulated stage is a PANI radical and an XG radical consolidated into XG-g-PANI. The chemical structure of the copolymer is reported to have been confirmed by IR spectroscopy, but no such structure is presented in the manuscript. The fabricated copolymer sample was tested as a material for sensing ammonia vapour. Prototype sensors were found to show high response, even at room temperature, in the ammonia vapour concentration range of 1–100 ppb, as well as reasonable response and recovery times (on the order of 10–30 s), making this a promising sensing material.

**Scheme 12:**
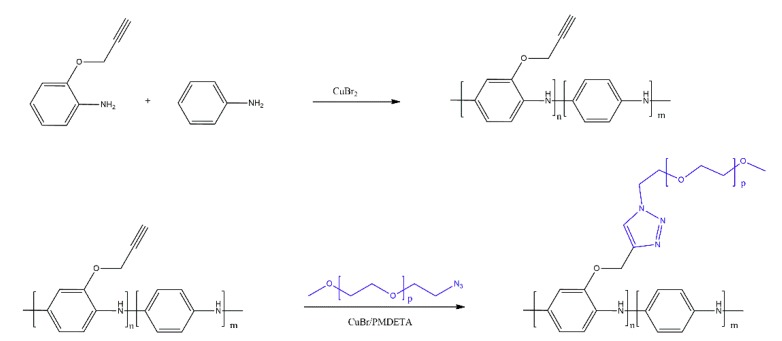
Grafting PEG chains on PANI by Bicak et al. [20].

Bicak et al. [20] proposed two methods of grafting poly(ethylene glycol) (PEG) chains on PANI, in order to bestow water solubility on the copolymer (Scheme 12). A combination of oxidative polymerisation and copper-catalysed azide–alkyne cycloaddition (CuAAC) click reaction is described. The method pertains to the reduction of the CuBr_2_ catalyst during the oxidative copolymerisation of aniline and aminophenyl propargyl ether to Cu(I) species, which catalyse the CuAAC reaction between thus-formed PANI with pendant alkyne groups and independently prepared azide-functionalised PEG both simultaneously and sequentially. The resulting copolymer, whose structure was confirmed by IR and NMR spectroscopy, was used as a sensor material for the detection of glucose. The prototype sensors showed linear response for solutions containing between 0.05 and 1 mmol/dm^3^ of glucose, with a limit of detection of 0.02 mmol/dm^3^ of glucose; these parameters were maintained even after 20 days of the sensor being stored in air, at −4 °C.

**Scheme 13:**
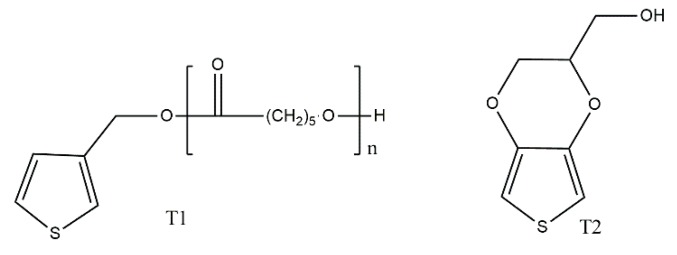
Macromonomer used by Molina et al. [21].

Another interesting approach to thiophene-based copolymers was reported by Molina et al. [21], who synthesised a macromonomer of polycaprolactone equipped with a 3-thienyl end group (**T1**), which was electrochemically polymerised with hydroxymethyl-3,4-ethylenedioxythiophene (**T2**) (Scheme 13). Expectedly, the **T1** macromonomer could not be polymerised, but **T2** readily produced a homopolymer. The structure of this homopolymer, as well as that of the copolymer of **T1** and **T2** was identified by IR spectroscopy. The homopolymer and copolymer were tested in terms of their biocompatibility, with the introduction of **T1** into the structure of the copolymer considerably reducing the cytotoxicity of the material, making it a potential candidate for producing implantable dopamine sensors.

**Scheme 14:**
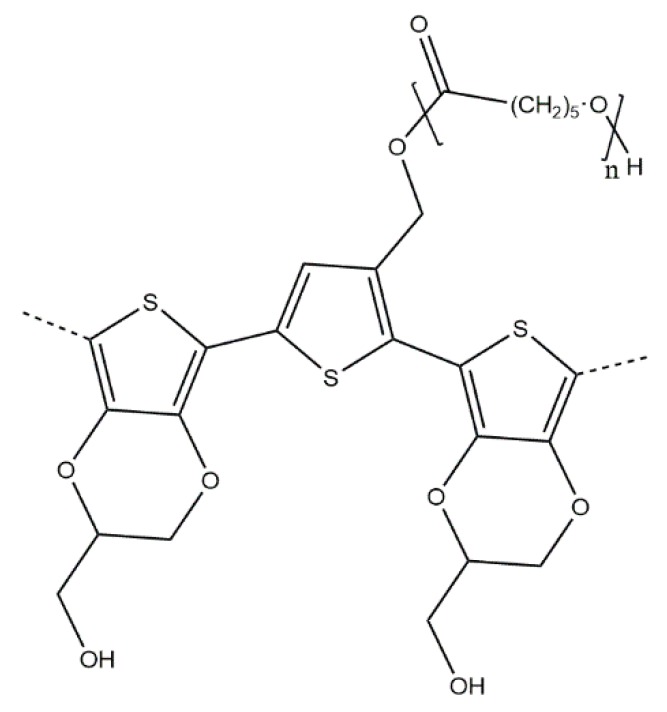
PPy-poly(Schiff base) copolymers, grafted with PEG by [22].

In another work Molina et al. [22] report the synthesis of PPy-poly(Schiff base) copolymers, grafted with PEG chains for the detection of serotonin. The synthesis of the graft copolymers was conducted sequentially, with a pyrrole-terminated PEG-bearing macromonomer first being synthesised chemically and later electrochemically polymerised in the presence of pyrrole to yield the final graft copolymer (Scheme 14). The structure of the copolymers was confirmed by IR spectroscopy, and bioactivity investigations showed that the copolymer had better biocompatibility than PPy (approx. 10–20% higher cell viabilities in comparison), as well as some antimicrobial activity. The serotonin-sensing properties were determined based on the peak maximum current density, as determined by differential pulse voltammetry, yielding serotonin detection limits of 0.04 and 0.07 μM for PPy and the investigated graft copolymer, respectively.

### 2.4. Graft Copolymers for Optoelectronic Applications

**Scheme 15:**
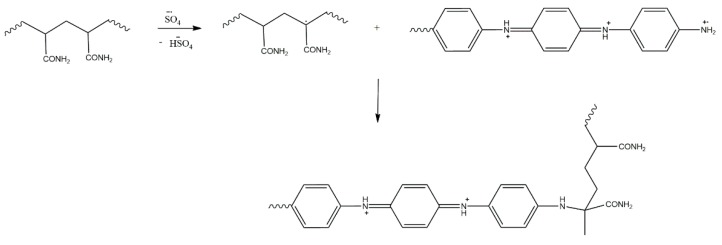
Grafting PAM which PANI by Smirnov et al. [23].

An interesting approach to graft copolymers is reported by Smirnov et al. [23], who produced fibrous mats for supercapacitor applications by electrospinning polyacrylamide (PAM), onto which PANI was grafted (Scheme 15). The structure of the copolymer was confirmed by IR spectroscopy, although only qualitative analysis is included in the report. Instead, although Smirnov et al. did employ several co-monomer ratios during their investigations, they assumed complete conversion of both co-monomers and their total inclusion in the copolymer, and report copolymer compositions based on the co-monomer feed ratios. It was found that by increasing the aniline to acrylamide ratio the fibre diameters could be decreased from 569 nm (for 20:80 aniline/acrylamide ratio) to 248 nm (40:60 ratio), but eventually (at ratios above 40:60) the resultant copolymer solutions became unfeasible for electrospinning, because they contained residual crystalline particles from PANI and were only suitable for electrospraying. The resultant fibrous mats were investigated in terms of their capacitance, showing at most 102 F/g (measured at 0.3 A/g), evidencing that this graft copolymer class consists of promising materials for supercapacitors.

**Scheme 16:**
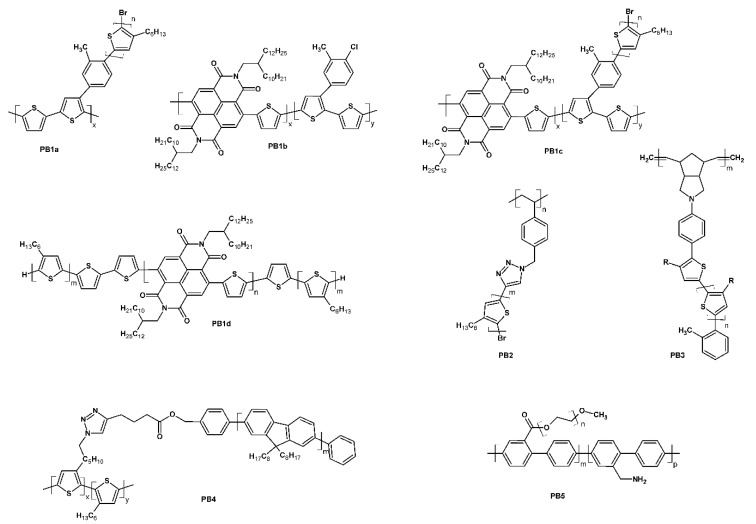
Structures of recently reported graft conjugated systems.

Wang et al., who earlier reported the synthesis of a series of conjugated graft copolymers [24,25,26,27] consisting of different conjugated cores, with poly(3-hexylthiophene) (P3HT) arms being grafted onto thiophene (Th) moieties of the cores, recently investigated a number of these copolymers (Scheme 16 **PB1a**–**d**) in prototype organic field-effect transistors (OFETs) and organic solar cells (OSCs) [28]. The performance of most of these donor–acceptor (D–A) copolymers in OFET configurations was relatively poor, exhibiting low ON/OFF current ratios and acceptable to extremely high threshold voltages (in the range of ~2–60 V). The former may be due to the balanced hole and electron mobility of these systems, as the best ON/OFF ratios of 1.14 × 10^2^ and 3.82 × 10^6^ were obtained respectively for the purely hole-transporting copolymer **PB1a** and for the mostly electron-transporting copolymer **PB1b**. The investigated compounds were mixed with both poly(3-hexylthiophene) and a fullerene derivative (PC_61_BM) and used in bulk hetero-junction (BHJ) OSCs, achieving power conversion efficiency (PCE) values of up to 2.27%. However, it is worth noting that in this case **PB1b** was used as a surfactant for the tried and true P3HT/PC_61_BM system. Unfortunately, the authors did not include the investigation of an OSC containing only P3HT/PC_61_BM and lacking the **PB1b** surfactant in the manuscript, making the impact of this addition on the power conversion efficiency of the OSC unclear.

Heinrich and Thelakkat [29] also used P3HT chains as arms, grafted onto a non-conjugated polystyrene (PS) core. They studied the impact of different structural parameters of this class of compounds on their properties, focusing on their charge carrier mobility. Interestingly, despite the inter-chain distances between P3HT arms resulting from a predetermined grafting scheme, the graft copolymers were shown to be crystalline, similar to linear P3HTs. The authors investigated PS-graft-P3HT copolymers (Scheme 16 **PB2**) in OFET configurations and compared them to the performance of pure linear P3HT references, achieving comparable results. A strong correlation of the charge carrier mobility and ON/OFF current ratio with the length of the P3HT arms was also observed. Although the ON/OFF current ratios achieved for the graft copolymers were comparable with those achieved for P3HT, that the number-average molecular weights of the copolymers were many times higher than those of P3HTs showing similar ON/OFF current ratios. As such, even though similar performance may be achieved, the processing of the copolymers may yet prove to be a significant limiting factor for the application of these new materials.

Another graft system equipped with P3HT arms was reported by van As et al. [30], who used P3HT chains capped with norbornene (NB) moieties as macromolecular monomers, to assemble a non-conjugated core through ring-opening metathesis polymerisation in a “grafting-through” process. The synthesised norbornene-functionalised P3HTs and graft polymers (**PB3**) were investigated in comparison with commercially available P3HT and linear P3HT samples of different molecular weight. The authors further investigated the performance of all systems in BHJ OSC configuration, using a mixture of the polymer and fullerene derivative as the active layer, reporting PCEs in the range of 0.3–2.3%. The best performance was observed for commercially available P3HT, with systems with a higher molecular weight generally showing higher PCEs. Although OSCs utilising the synthesised graft polymers showed slightly lower PCEs than those using linear P3HTs, they showed noticeably higher built-in voltages than devices based on linear P3HTs.

**Scheme 17:**
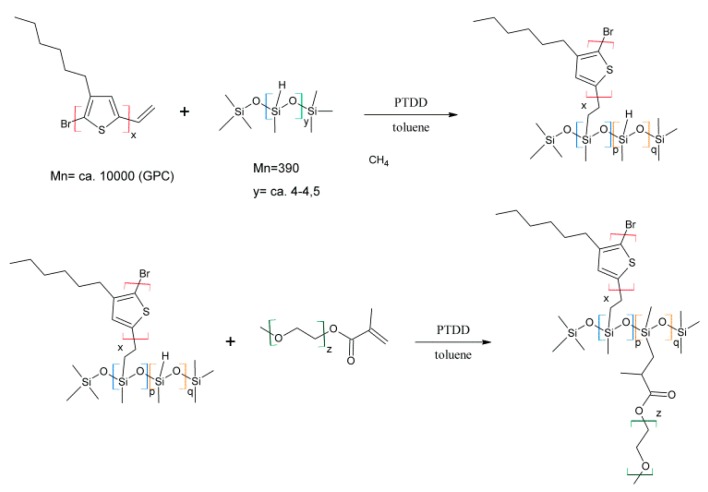
Synthesis of polysiloxanes grafted with PEG and P3HT [31].

Polysiloxanes grafted with both non-conjugated (PEG) and conjugated (P3HT) chains have been reported in our earlier works. These species were synthesised using hydrosilylation methods. This method depends on the grafting of co-monomers to the polysiloxane backbone through vinyl end groups (Scheme 17). Our copolymers show major spectroelectrochemical features similar to those of P3HT, but also feature improved doping/dedoping reversibility. Apart from improved stability and doping/dedoping reversibility, these graft copolymers were employed in prototype solar cells, showing power conversion efficiency values up to 2.11%—more than was achieved for solar cells containing the same amount of P3HT [31]. As such, this discovery can be considered an improvement in terms of device cost-efficiency, allowing lower amounts of P3HT to be used for their manufacture, rather than in terms of improved performance. It is worth noting that the obtained copolymers show lower conductivity in the undoped state than P3HT [32].

### 2.5. Graft Copolymers for Specialised Applications

**Scheme 18:**
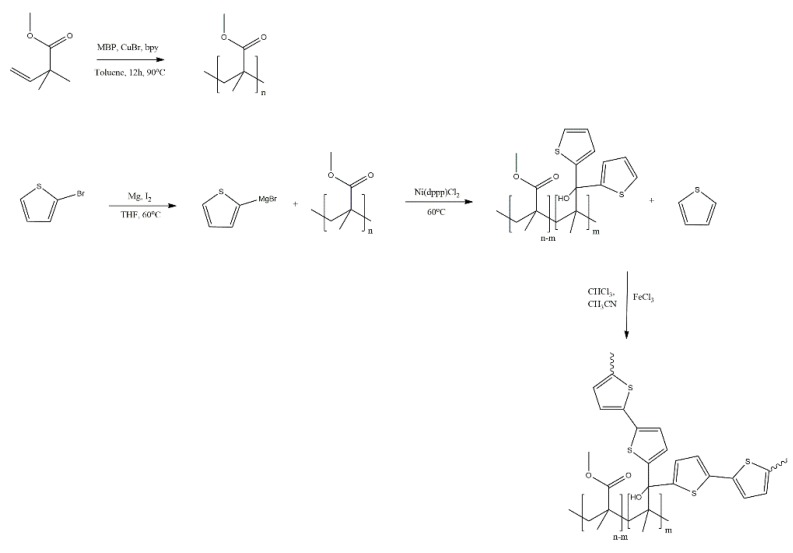
Grafting of polythiophene (PTh) onto poly(methyl methacrylate) (PMMA) [33].

Massoumi and Jaymand [33] report the grafting of polythiophene (PTh) onto poly(methyl methacrylate) (PMMA), performed both chemically and semi-electrochemically, as well as electrospinning of the copolymers with gelatine to produce conductive nanofibres (Scheme 18). Both PTh samples and the copolymers were investigated in terms of their electrochemical response and conductivity, yielding similar responses and slightly lower conductivity values for the copolymers that contained PMMA. Interestingly, the electrospun nanofibres showed conductivity lower by roughly two orders of magnitude than PTh and the copolymers. Although this may be the result of the incorporation of gelatine, no investigation of the doping level of the samples was conducted, and these differences in conductivity may stem from differences in the degree of doping. The authors perceive their nanofibres as promising materials for tissue engineering scaffold applications.

**Scheme 19:**
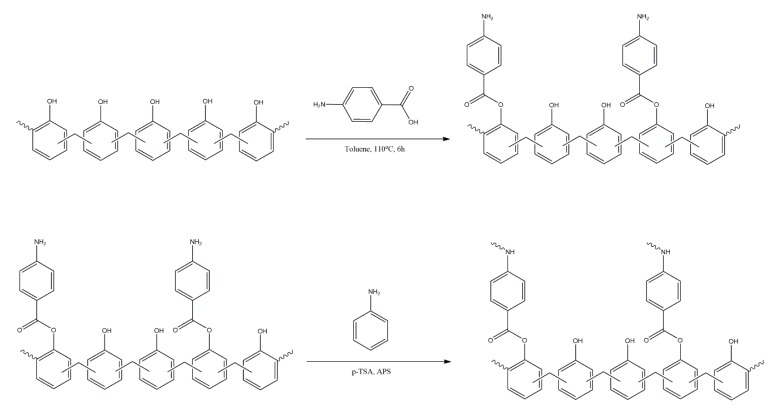
Grafting PANI onto novolacs [34].

In turn, Rezaei et al. [34] focused on the development of a new adhesive, by grafting PANI onto novolacs (Scheme 19). Interestingly, grafting was performed by treating the hydroxyl groups of novolacs with *p*-aminobenzoic acid, introducing amine groups into the novolac chains, which were used as grafting sites for PANI. The structure of the copolymers was confirmed by IR and NMR spectroscopy, although no quantitative investigation, particularly into the grafting density, was included in the manuscript. In terms of the mechanical properties, the copolymers showed properties similar to those of novolacs, with aniline units curiously causing a decrease in both the tensile strength and elasticity (elongation at break) of the materials. Interestingly, grafting PANI onto novolacs appeared not to influence the conductivity of PANI significantly, with the copolymers and pure PANI showing conductivity values on the same order of magnitude. As such, the copolymers are an interesting material choice for conductive adhesives, with a multitude of potential applications.

**Scheme 20:**
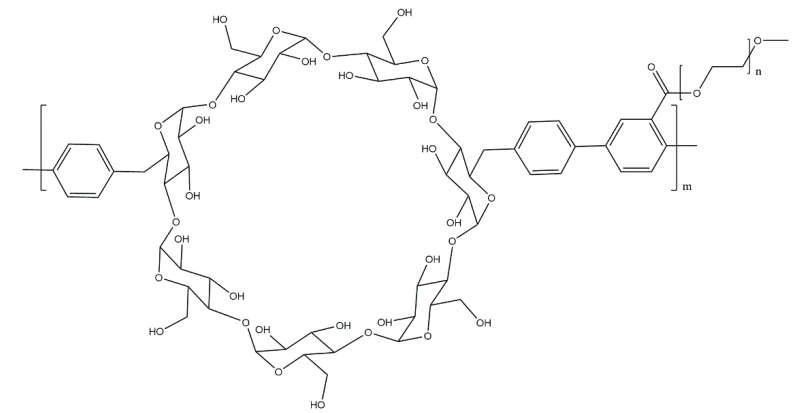
Poly(*p*-phenylene-β-cyclodextrin)-graft-PEG synthesised by Barlas et al. [35].

Barlas et al. [35] report the use of a fluorescent poly(*p*-phenylene) derivative, equipped with cyclodextrin units in the main chain and with poly(ethylene glycol) grafts (Scheme 20), as a novel material for tumour cell imaging and radiotherapy. This previously synthesised and investigated molecule was then conjugated with Au nanoparticles, and these nanoconjugates were investigated for their cytotoxic and cytostatic effects, with a concentration of 100 μg/mL being found as the minimum required for producing a decrease in cell viability. Fluorescence imaging studies showed that the derivative containing cyclodextrin units resulted in a noticeably higher quality of the image than was previously achieved for poly(*p*-phenylene) equipped with PEG grafts, making this material a promising candidate for therapeutic applications.

Controlled anti-cancer drug delivery is a relatively new field of application for conjugated polymer materials, often relying on doping/dedoping to charge a polymer matrix with a drug and release it upon the application of stimuli [13]. Guler et al. [36] recently reported an interesting graft polymer for drug delivery applications, consisting of a conjugated core and non-conjugated arms. However, in this case the drug loading/release mechanism is based on pH rather than doping, with the conjugated polyparaphenylene core used both as scaffold for functional groups (acting as bonding sites for loading drug molecules into the matrix) and as a sensitising agent for radiotherapy. The polymer/drug composite was shown to be more effective than the pure drug in bio-conjugation studies, and significantly more effective in radiotherapy studies.

**Scheme 21:**
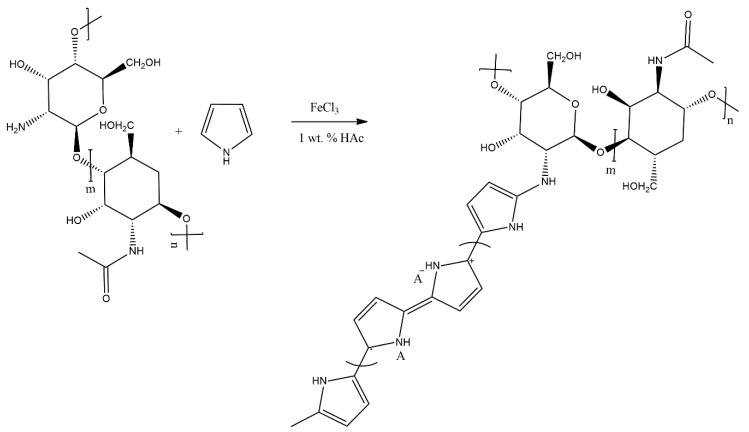
Grafting chitosan with PPy [37].

Cabuk et al. [37] have also undertaken the subject of grafting chitosan with conducting polymers, but opted to graft it with PPy (Scheme 21). The copolymer was investigated in terms of its electrokinetic properties in aqueous media, showing a shift of the ζ-potential of the copolymer towards more positive values than in the case of PPy. Suspensions of the copolymer in silicone oil were found to show some electrorheological activity, exhibiting viscoelastic behaviour and showing a reversible nonlinear deformation when placed in an electric field. As such, the graft copolymer may have potential as a material for vibration damping applications.

**Scheme 22:**
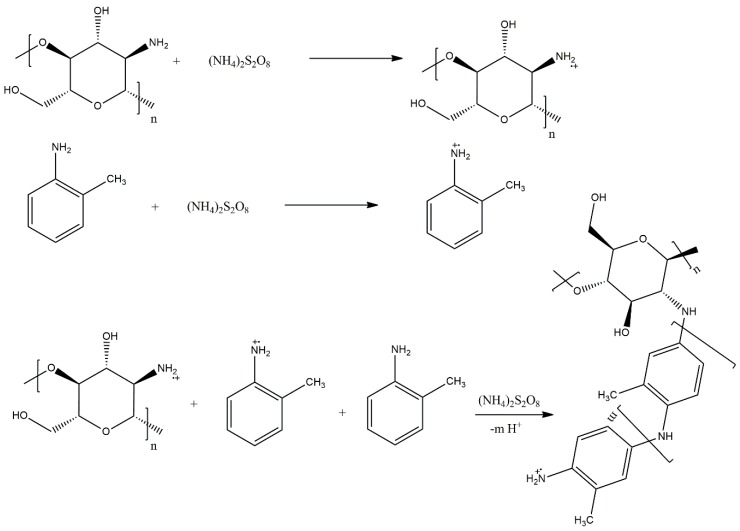
Grafting chitosan with poly(2-methylaniline) [38].

A novel application of graft copolymers, as materials for wastewater treatment, is reported by Abd El-Salam et al. [38], who utilised a graft system composed of poly(2-methylaniline) and chitosan (Scheme 22). Investigation of the wastewater treatment potential of both “parent” homopolymers and the graft copolymer showed that while poly(2-methylaniline) had very poor coliform removal properties and chitosan showed acceptable performance, the copolymer was found to be approximately twice as efficient as chitosan at removing coliform bacteria, making it a promising material for wastewater treatment. The authors attribute this increased efficiency to the presence of more function sites on the surface of the graft copolymer.

**Scheme 23:**
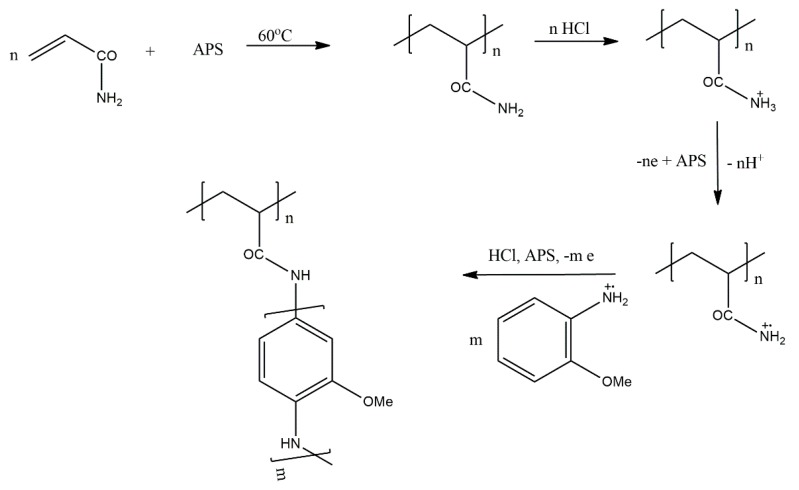
Copolymerisation of 2-methoxyaniline onto PAM.

PAM-graft-poly(2-methoxyaniline) was synthesised [39] by the oxidative chemical grafting copolymerisation of 2-methoxyaniline onto PAM (Scheme 23). The grafting was performed in aqueous HCl using ammonium peroxydisulphate as an oxidising agent. The efficiency of the prepared polymeric samples to remove some metal ions from contaminated water was investigated, removing 27.9%, 99.4% and 51.1% of manganese (II), lead (II) and chromium (II), respectively, from a solution containing 2 mg/mL of each ion. In light of these results, the obtained graft copolymer can be considered a selective adsorbent for Pb (II) ions present in a mixture with Mn (II) and Cr (II) ions.

Graft copolymers can also be used as anti-corrosion coatings, as exemplified by the report by Babaladimath et al., who chose PANI grafted onto xanthan gum as a potential coating material [40] (analogous to the reaction shown in Scheme 11). The grafting was achieved by polymerisation of aniline in the presence of xanthan gum, under acidic conditions, employing microwave irradiation. In terms of anti-corrosion properties, although the report seems to have some minor data conflicts, Tafel polarisation experiments showed that the graft copolymer significantly reduced the corrosion currents in comparison with bare aluminium surfaces. A similar conclusion can be drawn from electrochemical impedance spectroscopic measurements, where the samples coated with the graft copolymer showed a significantly higher charge transfer resistance than bare aluminium surfaces. As such, the graft copolymer could be a promising and specialised anti-corrosive coating material.

## 3. Summary and Future Outlook

Of the reviewed articles, the vast majority (28 reports) focus on specific applications of either newly produced macromolecules or known macromolecules not considered beforehand in particular applications. Among the selected works, the development and application of hydrogels seems to have recently attracted the interest of researchers dealing with copolymers (six reports), owing mostly to their biomedical applications. Simultaneously, the once extremely popular subjects of optoelectronic (six reports) and drug delivery applications seem to have declined slightly. Among the specialised applications, we can see the development in wastewater treatment methods and its emerging interest in new copolymer materials (two reports). Although there are some reports focusing more on the mechanical aspects of the copolymers (two reports), dealing with adhesives or the manufacture of copolymer fibres, the increased conductivity of the copolymers, stemming from the inclusion of conjugated repeat units in their structure, may only find niche applications due to the increased cost of producing such materials, as opposed to traditional homopolymers. Although not as recent as some of the others, one particularly interesting application is the use of conjugated unit-bearing copolymers in vibration damping, due to their electrorheological properties.

In terms of structural investigation of the copolymers, the methodology is virtually identical to the one employed for the study of homopolymers, leaning heavily on IR and NMR spectroscopy (Table 1). One key difference between the two is the use of gravimetric methods to study the amount of grafts introduced into a homopolymer sample. Although gravimetry can provide reliable information about the composition of the produced copolymer, it cannot be used to discern between the different species in the sample. As such, the technique is heavily susceptible to the effects of impurities present in the samples. In the case of preparing graft copolymers, this may include non-grafted chains of either of the “parent” homopolymers, reagents occluded within the polymer matrix, etc. Consequently, this technique should be used only with thorough care, as in the model case of Hatamzadeh et al. [9]. It is worth noting that recently, only a few works have even attempted to quantitatively investigate the composition of the copolymers they were dedicated to, making the usefulness of the results presented in works lacking such studies uncertain.

In terms of the approach to the grafting mechanism, grafting “from” appears to be the most commonly used approach, with 15 works utilising it. In terms of the sheer synthetic pathway, it is worth mentioning the frequent use of oxidative polymerisation, particularly mediated by (NH_4_)_2_S_2_O_8_ (APS)—especially in the case of grafting PANI. Apart from those, there are few reports using click chemistry and polymerising macromonomers, as well as mention of introducing grafts via electrochemical methods. Nevertheless, the more straightforward and cost-efficient a method, the more works tend to employ it. This is due to the relatively young age of the field; most reported applications are general in nature and can rely on various copolymer materials, with highly specialised application–material (or material class) pairs yet to emerge.

Based on the trends presented in this mini review, we can expect future works to slowly be shifting their focus away from general and basic investigations into the properties of conjugated graft copolymers and towards reporting new and specialised applications that can utilise the properties specific to each copolymer class. The emergence of these specialised applications and their need for high-performance materials is expected to promote developing and manufacturing materials tailored for those particular applications.

## Figures and Tables

**Table 1:** Summary of the structural identification methods used in the reviewed works.

Copolymer	Copolymer Structural Identification	Study of Copolymer Molecular Weight	Quantitative Composition of the Copolymer	Literature
Poly(2-hydroxyaniline)-co-chitosan	IR	No	No	[7]
PANI-graft-PSMA	IR	No	No	[8]
PS-graft-PPy	IR	GPC	Yes	[9]
PANI-graft-PVC	IR	No	No	[10]
QCS-graft-PANI	IR, gravimetry	No	Yes	[11]
Chitosan-graft-PANI	IR	No	No	[12]
Dextran-graft-(aniline tetramer)-graft-(4-formylbenzoic acid)	IR, NMR	No	No	[15]
(Oxidised hyaluronic acid)-graft-AT	IR	No	No	[16]
Gelatine-graft-PANI	IR	No	No	[18]
XG-graft-PANI	IR, gravimetry	No	No	[19]
PEG-graft-PANI	IR, NMR	No	No	[20]
PTh-graft-PCL	IR	No	No	[21]
PPy-poly(Schiff base) copolymers-graft-PEG	IR	No	No	[22]
PAM-graft-PANI	IR	No	No	[23]
Conjugated cores-graft-P3HT	Prior works	SEC	n/a	[28]
PS-graft-P3HT	NMR, IR	MALDI-TOF, SEC	No	[29]
PolyNB-graft-P3HT	NMR	MALDI-TOF, GPC	No	[30]
Polysiloxanes-graft-PEG-graft-P3HT	NMR, IR	No	Semi	[31]
PANI-graft-novolac	NMR (macromonomer only), IR	No	No	[34]
Poly(p-phenylene-β-cyclodextrin)-graft-PEG	Prior works	No	No	[35]
Poly(p-phenylene)-graft-PEG	Prior works	GPC	No	[36]
Chitosan-graft-PPy	Prior works, Elemental analysis	No	No	[37]
Chitosan-graft-poly(2-methylaniline)	IR	No	No	[38]
PAM-graft-poly(2-methoxyaniline)	IR, NMR, Gravimetry	No	No	[39]
PEG-graft-PANI	IR, Gravimetry	No	No	[40]

## Abbreviations

APS	ammonium peroxydisulphate
AT	aniline tetramer
BHJ	bulk hetero-junction
CuAAC	copper-catalysed azide–alkyne cycloaddition
IPN	interpenetrating polymer network
IR	infrared
NMR	nuclear magnetic resonance
OFET	organic field-effect transistor
OSC	organic solar cell
P3HT	poly(3-hexylthiophene)
PAM	polyacrylamide
PANI	polyaniline
PANI-g-PSMA	polyaniline-graft-poly(styrene-alt-maleic anhydride)
PCE	power conversion efficiency
PEDOT	poly(3,4-ethylenedioxythiophene)
PEG	poly(ethylene glycol)
PMMA	poly(methyl methacrylate)
PPy	polypyrrole
PS	polystyrene
PSMA	poly(styrene-alt-maleic anhydride)
PTh	polythiophene
PVC	poly(vinyl chloride)
QCS	quaternised chitosan
QCS-g-PANI	quaternised chitosan-graft-polyaniline
THF	tetrahydrofuran
XG	xanthan gum
